# Supplementary material for: Comprehensive Analysis of Risk Factors for Periodontitis Focusing on the Saliva Microbiome and Polymorphism
Source: Int J Environ Res Public Health. 2021 Jun 14;18(12):6430. doi: 10.3390/ijerph18126430 (PMC8296229; doi:10.3390/ijerph18126430)
Supplement: Supplementary file 1 [file ijerph-18-06430-s001.zip › supplemental figure caption.pdf]

Figure S1. Species richness estimated by Shannon index (a), Simpson's index (b), Chao1 (c), and abundance-based coverage estimator (ACE) (d) based on operational taxonomic unit (OTU) abundance for periodontitis and control groups.
