# Supplementary material for: Comprehensive Analysis of Risk Factors for Periodontitis Focusing on the Saliva Microbiome and Polymorphism
Source: Int J Environ Res Public Health. 2021 Jun 14;18(12):6430. doi: 10.3390/ijerph18126430 (PMC8296229; doi:10.3390/ijerph18126430)
Supplement: Supplementary file 1 [file ijerph-18-06430-s001.zip › Table S1.pdf]

Table S1. Difference in genotypic frequencies between periodontitis and control groups.

Gene	SNPs	Gene polymorphisms	Periodontitis group n =11	Control group n = 11	P[a]
KCNQ5	rs9446777	AA	8 (72.7)[b]	7 (63.6)	0.587
		AG	3 (27.3)	3 (27.3)	
		GG	0 (0.0)	1 (9.1)	
TENM2	rs4242220	TT	7 (63.6)	8 (72.7)	0.420
		TG	4 (36.4)	2 (18.2)	
		GG	0 (0.0)	1 (9.1)	
NPY	rs2521634	GG	6 (54.5)	9 (81.8)	0.118
		AG	5 (45.5)	1 (9.1)	
		AA	0 (0.0)	1 (9.1)	
ADGRE1	rs3826782	GG	4 (36.4)	6 (54.5)	0.392
		GA	7 (63.6)	5 (45.5)	
		AA	0 (0.0)	0 (0.0)	
TRA	rs3811273	AA	4 (36.4)	8 (72.7)	0.147
		AG	5 (45.5)	3 (27.3)	
		GG	2 (18.2)	0 (0.0)	
C1QTNF7	rs4074082	TT	10 (90.9)	8 (72.7)	0.586
		TC	1 (9.1)	3 (27.3)	
		CC	0 (0.0)	0 (0.0)	
TSNARE	rs9772881	AA	7 (63.6)	10 (90.9)	0.282
		GA	3 (27.3)	1 (9.1)	
		GG	1 (9.1)	0 (0.0)	
HPVC1	rs10232172	GG	9 (81.8)	9 (81.8)	1.000
		GT	2 (18.2)	2 (18.2)	
		TT	0 (0.0)	0 (0.0)	
PKP2	rs6488099	AA	11 (100)	11 (100)	NA
		AG	0 (0.0)	0 (0.0)	
		GG	0 (0.0)	0 (0.0)	
FHOD3	rs8094794	CC	7 (63.6)	9 (81.8)	0.635
		CT	4 (36.4)	2 (18.2)	
		TT	0 (0.0)	0 (0.0)	
RF00026	rs733048	GG	4 (36.4)	4 (36.4)	0.856
		AG	5 (45.5)	4 (36.4)	
		AA	2 (18.2)	3 (27.3)	

CDKL1	rs3783412	GG	4 (36.4)	1 (9.1)	0.141
		AG	5 (45.5)	4 (36.4)	
		AA	2 (18.2)	6 (54.5)	
FBXO38	rs10043775	TT	8 (72.7)	7 (63.6)	0.875
		CT	2 (18.2)	3 (27.3)	
		CC	1 (9.1)	1 (9.1)	
TBC1D1	rs10010758	TT	8 (72.7)	7 (63.6)	0.875
		CT	2 (18.2)	3 (27.3)	
		CC	1 (9.1)	1 (9.1)	
TENM2	rs6885116	AA	10 (90.9)	8 (72.7)	0.586
		AG	1 (9.1)	3 (27.3)	
		GG	0 (0.0)	0 (0.0)	
MTND1P5	rs16870060	GG	7 (63.6)	9 (81.8)	0.484
		GT	3 (27.3)	2 (18.2)	
		TT	1 (9.1)	0 (0.0)	
ASH1L	rs13373934	AA	11 (100)	11 (100)	NA
		AG	0 (0.0)	0 (0.0)	
		GG	0 (0.0)	0 (0.0)	
IL1A	rs17561	CC	9 (81.8)	9 (81.8)	1.000
		CA	2 (18.2)	2 (18.2)	
		AA	0 (0.0)	0 (0.0)	
IL1A	rs1800587	AA	9 (81.8)	9 (81.8)	1.000
		AG	2 (18.2)	2 (18.2)	
		GG	0 (0.0)	0 (0.0)	
IL1B	rs1143627	AA	1 (9.1)	1 (9.1)	1.000
		AG	6 (54.5)	6 (54.5)	
		GG	4 (36.4)	4 (36.4)	
IL1B	rs16944	GG	1 (9.1)	1 (9.1)	1.000
		AG	6 (54.5)	6 (54.5)	
		AA	4 (36.4)	4 (36.4)	
IL1B	rs1143633	GG	3 (27.3)	2 (18.2)	0.842
		CG	5 (45.5)	5 (45.5)	
		CC	3 (27.3)	4 (36.4)	
IL1B	rs1143634	GG	10 (90.9)	10 (90.9)	1.000
		AG	1 (9.1)	1 (9.1)	
		AA	0 (0.0)	0 (0.0)	
NIN (Intronic)	rs12883458	TT	6 (54.5)	9 (81.8)	0.361

		CT	5 (45.5)	2 (18.2)	
		CC	0 (0.0)	0 (0.0)	
WNT5A (109Kb)	rs11925054	GG	11 (100)	11 (100)	NA
		GT	0 (0.0)	0 (0.0)	
		TT	0 (0.0)	0 (0.0)	
PTGFRN	rs6589	TT	11 (100)	11 (100)	NA
		CT	0 (0.0)	0 (0.0)	
		CC	0 (0.0)	0 (0.0)	
TNS1	rs1004814	CC	4 (36.4)	3 (27.3)	0.149
		CT	6 (54.5)	3 (27.3)	
		TT	1 (9.1)	5 (45.5)	
ANKRD28	rs983600	GG	11 (100)	11 (100)	NA
		AG	0 (0.0)	0 (0.0)	
		AA	0 (0.0)	0 (0.0)	
C4orf21	rs17669218	GG	11 (100)	11 (100)	NA
		AG	0 (0.0)	0 (0.0)	
		AA	0 (0.0)	0 (0.0)	
VARS	rs707926	GG	3 (27.3)	5 (45.5)	0.659
		AG	7 (63.6)	5 (45.5)	
		AA	1 (9.1)	1 (9.1)	
PRRC2A	rs1046080	CC	11 (100)	11 (100)	NA
		AC	0 (0.0)	0 (0.0)	
		AA	0 (0.0)	0 (0.0)	
DGKB	rs1997040	AA	8 (72.7)	9 (81.8)	1.000
		AC	3 (27.3)	2 (18.2)	
		CC	0 (0.0)	0 (0.0)	
RFC2	rs1805395	TT	11 (100)	11 (100)	NA
		TC	0 (0.0)	0 (0.0)	
		CC	0 (0.0)	0 (0.0)	
MYOF	rs11187393	GG	11 (100)	11 (100)	NA
		AG	0 (0.0)	0 (0.0)	
		AA	0 (0.0)	0 (0.0)	
OR11H6	rs17277228	TT	7 (63.6)	7 (63.6)	1.000
		CT	4 (36.4)	4 (36.4)	
		CC	0 (0.0)	0 (0.0)	
ZFYVE1	rs12931	CC	5 (45.5)	4 (36.4)	0.904
		AC	5 (45.5)	6 (54.5)	

		AA	1 (9.1)	1 (9.1)	
ADPGK	rs9460	GG	10 (90.9)	11 (100)	1.000
		AG	0 (0.0)	0 (0.0)	
		AA	1 (9.1)	0 (0.0)	
TAF1C	rs3743642	GG	9 (81.8)	10 (90.9)	0.591
		AG	1 (9.1)	1 (9.1)	
		AA	1 (9.1)	0 (0.0)	
PSMD13	rs1045288	AA	9 (81.8)	8 (72.7)	0.357
		AG	1 (9.1)	3 (27.3)	
		GG	1 (9.1)	0 (0.0)	
Vitamin D receptor (VDR)	rs731236	AA	9 (81.8)	10 (90.9)	1.000
		AG	2 (18.2)	1 (9.1)	
		GG	0 (0.0)	0 (0.0)	
IL10	rs6667202	CC	11 (100)	11 (100)	NA
		AC	0 (0.0)	0 (0.0)	
		AA	0 (0.0)	0 (0.0)	
GLT6D1	rs1537415	GG	8 (72.7)	2 (18.2)	0.035
		GC	2 (18.2)	7 (63.6)	
		CC	1 (9.1)	2 (18.2)	
ANRIL	rs1333048	AA	4 (36.4)	3 (27.3)	0.867
		AC	3 (27.3)	4 (36.4)	
		CC	4 (36.4)	4 (36.4)	
VDR	rs10735810	CC	4 (36.4)	3 (27.3)	0.170
		AC	3 (27.3)	7 (63.6)	
		AA	4 (36.4)	1 (9.1)	
TLR1	rs5743611	CC	11 (100)	11 (100)	NA
		CG	0 (0.0)	0 (0.0)	
		GG	0 (0.0)	0 (0.0)	
IFI16	rs6940	AA	6 (54.5)	7 (63.6)	0.584
		AT	4 (36.4)	4 (36.4)	
		TT	1 (9.1)	0 (0.0)	
IFI17	rs1057028	TT	5 (45.5)	5 (45.5)	0.580
		AT	5 (45.5)	6 (54.5)	
		AA	1 (9.1)	0 (0.0)	
DEFB1	rs1800972	GG	8 (72.7)	7 (63.6)	1.000
		CG	3 (27.3)	4 (36.4)	
		CC	0 (0.0)	0 (0.0)	

DEFB1	rs11362	CC	3 (27.3)	4 (36.4)	0.540
		CT	5 (45.5)	6 (54.5)	
		TT	3 (27.3)	1 (9.1)	
LTF	rs1126478	TT	7 (63.6)	4 (36.4)	0.231
		CT	4 (36.4)	5 (45.5)	
		CC	0 (0.0)	2 (18.2)	
LTF	rs1126477	CC	1 (9.1)	6 (54.5)	0.068
		CT	7 (63.6)	3 (27.3)	
		TT	3 (27.3)	2 (18.2)	

[a] Chi-squared test
[b] N (%)
