# Supplementary figures and images for: Comprehensive Analysis of Risk Factors for Periodontitis Focusing on the Saliva Microbiome and Polymorphism
Source: Int J Environ Res Public Health. 2021 Jun 14;18(12):6430. doi: 10.3390/ijerph18126430 (PMC8296229; doi:10.3390/ijerph18126430)

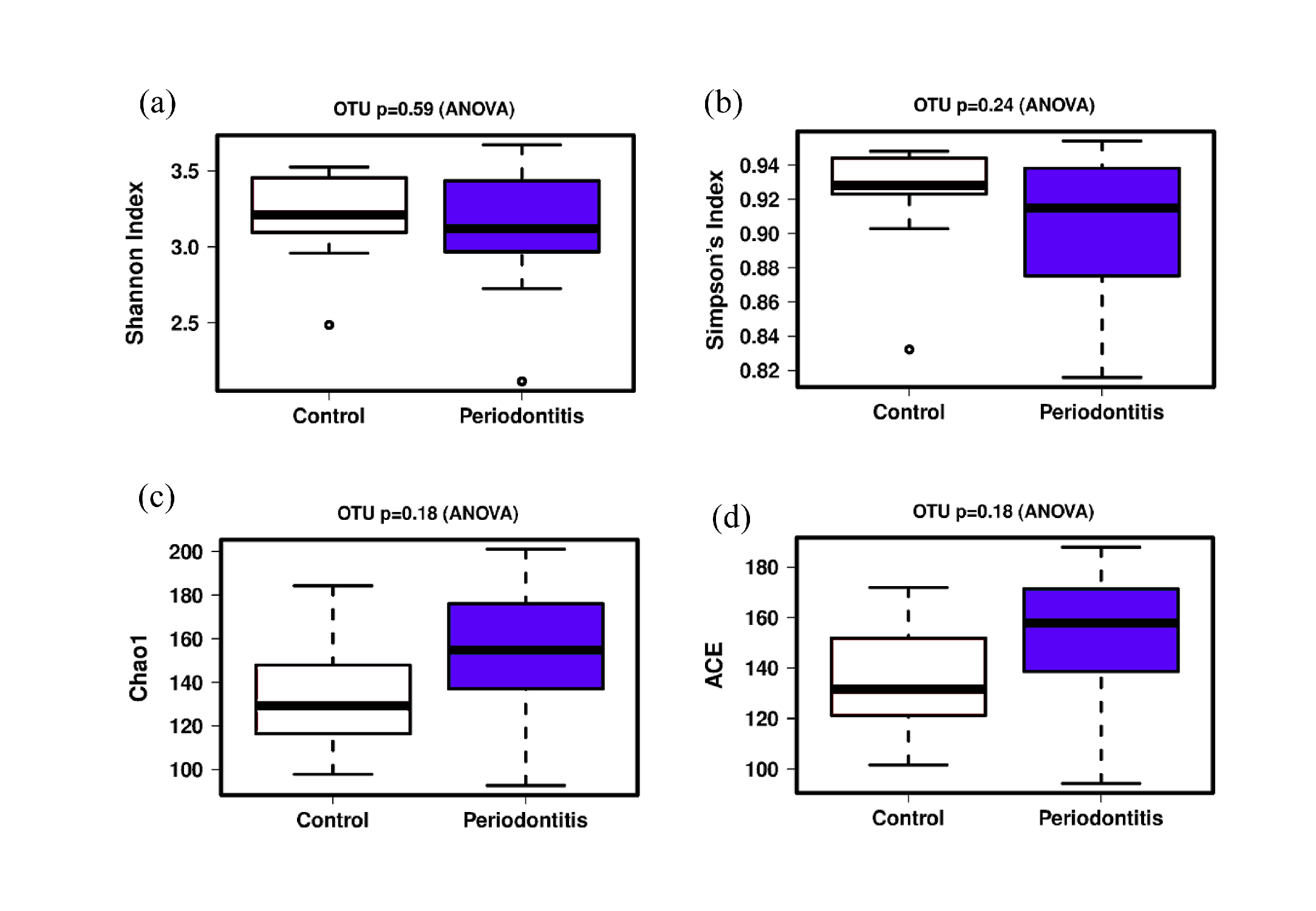

Supplement: Supplementary file 1 [file ijerph-18-06430-s001.zip › figure S1 TIFF.tif]
